# Immuno-physiological effects of dietary reishi mushroom powder as a source of beta-glucan on Rohu, *Labeo rohita* challenged with *Aeromonas veronii*

**DOI:** 10.1038/s41598-023-41557-9

**Published:** 2023-09-05

**Authors:** Tutul Kumar Saha, Mariom, Tanvir Rahman, Mohammad Moniruzzaman, Taesun Min, Zakir Hossain

**Affiliations:** 1 Department of Fisheries Biology and Genetics, Faculty of Fisheries, Bangladesh Agricultural University, Mymensingh, 2202 Bangladesh; 2 Department of Aquaculture, Faculty of Fisheries, Bangladesh Agricultural University, Mymensingh, 2202 Bangladesh; 3 Department of Animal Biotechnology, Jeju International Animal Research Center, Jeju National University, Jeju, 63243 Republic of Korea; 4 Department of Animal Biotechnology, Bio-Resources Computing Research Center, Jeju National University, Jeju, 63243 Republic of Korea

**Keywords:** Interferons, Interleukins, Lymphokines, Tumour-necrosis factors, Immunosuppression, Bacterial infection, NK cells, Immunological models, Enzymes, Immunogenetics, Immunopathogenesis, Nutrition, Animal physiology, Ichthyology, Adaptive immunity, Autoimmunity, Cytokines, Haematopoiesis

## Abstract

Beta-glucans have immense potential to stimulate immune modulation in fish by being injected intramuscularly, supplemented with feed or immersion routes of administration. We studied how supplementing *Labeo rohita*’s diet with reishi mushroom powder containing beta-glucan influenced immunological function. A supplemented diet containing 10% reishi mushroom powder was administered for 120 days. Afterwards, analyses were conducted on different immunological parameters such as antioxidants, respiratory burst, reactive oxygen species (ROS), alternative complement activity, and serum immunoglobulin, which resulted significant increases (*p* < 0.05; *p* < 0.01) for the reishi mushroom-fed immune primed *L. rohita*. Additionally, analyzing various hematological parameters such as erythrocytes and leukocytes count were assessed to elucidate the immunomodulatory effects, indicating positive effects of dietary reishi mushroom powder on overall fish health. Furthermore, the bacterial challenge-test with 1.92 × 10^4^ CFU/ml intramuscular dose of *Aeromonas veronii* showed enhanced disease-defending system as total serum protein and lysozyme activity levels accelerated significantly (*p* < 0.01). Nevertheless, reishi mushroom powder contained with beta-glucan ameliorated the stress indicating parameters like acetylcholinesterase (AChE), serum-glutamic pyruvic transaminase (SGPT) and serum-glutamic oxaloacetic transaminase (SGOT) enzyme activities results suggested the fish’s physiology was unaffected. Therefore, the results indicated that adding dietary reishi mushroom as a source of beta-glucan could significantly boost the immune responses in Rohu.

## Introduction

In recent days, immunostimulants have been widely regarded as valuable tools for improving the immune status of cultured organisms, particularly in aquatic living systems^1^. Beta-glucan, to be more specific, is a homopolysaccharide composed entirely of glucose molecules joined together via glycosidic bonds, and which is a promising immunostimulant used in aquaculture^2^. Beta-glucan is used by many different types of plants, seaweeds, yeast, fungi, mushrooms, bacteria, and to construct their cell walls. Consequently, the majority of research on beta-glucan has mainly focused on understanding its receptors and mechanism of action. In animals, beta-glucan is recognized and bound to by an endogenous receptor, resulting in increased resistance to infection and a stronger immune response to infectious pathogens found in fish and shellfish^2^.

Mushrooms have been appreciated as health-promoting agents for hundreds of years as its one of the particular components found is beta-glucan, which consists mainly of beta-d-glucose and is found in the walls of fungi^3^. Numerous studies reported that the immune system is strengthened by beta-glucan, making it more capable of fighting off microbial, viral, fungal, and parasitic invaders^4^. It is also regarded that mushroom cell walls are densely packed with beta-glucans, which are linear and branching glucose polymers with beta-1,3 and beta-1,6 linkages, respectively^3^. A well-known form of mushroom used in Chinese medicine is the reishi mushroom (*Ganoderma lucidum*), which has a wide variety of pharmacologically active ingredients and special health advantages^4^. It has a number of crucial components. Particularly, the triterpenoids, polysaccharides and beta-glucan found in these mushrooms have health-promoting and therapeutic properties, including immunomodulatory, antioxidant, and hepatoprotective effects^5^. Numerous scholarly articles have underscored the advantageous use of reishi mushrooms for various fish species. For example, the administration of *G. lucidum* extract was found to enhance the immune response and disease resistance in Nile tilapia^6,7^ and orange-spotted grouper^8^. Moreover, it has been observed that the exopolysaccharides derived from the mycelial extract of *G. lucidum* have demonstrated significant efficacy in enhancing the activity of antioxidant enzymes and promoting the overall health and growth of red hybrid tilapia^9^ and nile tilapia^7^.

According to early research, beta-glucan has been shown in numerous fish studies to be an effective immunostimulant making it a promising implement for improving fish health and preventing disease in aquaculture^10,11^. For the assessment of its effect as an active immune booster, a series of parameters have been undertaken in various studies, namely, antioxidant activity^12^, serum immunoglobulin^13^, respiratory burst activity^14^, reactive oxygen species^15^, and different hematological parameters^16,17^ such as glucose, protein as well as blood cells count. Blood leukocytes are primarily responsible for the formation of additional cellular components of innate immune function. They secrete a wide variety of humoral substances that are capable of killing off abnormal and foreign (allogeneic or xenogeneic) cells immediately, including cationic antimicrobial peptides, complement components, lectins, cytokines, anti-inflammatory, and immune mediators like IL (interleukin)-10 and TGF-beta. The immune system as a whole benefits from the release of these substances into the bloodstream, epithelial fluid, and skin mucus^17^.

There has been extensive use of non-specific immunostimulants in the aquaculture industry, most likely due to a lack of understanding of the immunological response of fish and the ease with which they can be applied. As an immunostimulant, reishi mushroom has enormous potential in fish culture, disease management, and fish product development. This provides immunity against various fish pathogens as a prophylactic and disease management agent^5^. Correspondingly, it has also been indicated that they support the actions of other non-specific immune factors, such as serum protein^18^ and lysozyme^19^ activities to be able to prevent bacterial infection. For a long time, since considering the current study concept, the number of studies involving mushroom has increased for fish physiological and immunological responses^7–9^. However, our investigation provided particular knowledge on the dietary reishi mushroom as it exists a diverse array of pharmacologically active elements that possess distinct health benefits^5^. Specifically, this may produce a broodfish with a better immunological profile which may eventually create a chance of enhanced immunity in the future offspring. Therefore, reishi mushroom as a source of beta-glucan has been administrated as an immunostimulant that allows us to achieve our goal. Thereafter, the Indian major carp, *L. rohita* have been selected as a model organism as it faces the challenge of fish mortality due to bacterial diseases and also can be used as a model species for immunizing other farmed fish. In this present study, different stress-associated enzymatic activities have also been considered, including a neurotransmitter called acetylcholinesterase (AChE); glutamic oxaloacetic transaminase (GOT) and glutamic pyruvic transaminase (GPT): two different enzyme levels in serum were used to determine the presence of stress in fish. Finally, we evaluated the effects of dietary reishi mushroom on overall fish immunity through the bacterial challenge test, where we hypothesized that reishi mushroom powder has a positive impact on enhancing the immune system in Rohu carp without compromising the health status of the fish.

## Results

### Effects of reishi mushroom powder as an immunity enhancer

#### Hematological parameters

The blood parameters of the experimental fish were changed after feeding with the reishi mushroom diet for 4 months trial, as shown in Table 1. The blood hemoglobin (Hb) of fish provided with reishi mushroom diet was significantly greater than that of fish in the control group and the figure comprised with 13.56 ± 0.77 g/dl and 9.40 ± 0.61 g/dl, respectively. In the treated group, the number of RBCs was determined to be 3.39 ± 0.35 × 10^6^/µl, which was a significant (*p* < 0.01) increase when compared to the control group (2.34 ± 0.23 × 10^6^/µl). The WBCs count of the treatment was recorded as 8.26 ± 0.63 × 10^3^/µl, which was significantly greater than the control value, 7.17 ± 0.44 × 10^3^/µl. Additionally, the total serum protein value of reishi mushroom powder-treated fish was significantly different from the control value and found as 3.15 ± 0.32 g/dl and 2.66 ± 0.25 g/dl, respectively. However, blood glucose level variations were statistically insignificant.

**Table 1:** Haematological parameters of *L. rohita* (data have been presented as mean ± standard error).

Parameters	Control (mean ± S.E.)	Treatment (mean ± S.E.)
RBC (× 10^6^/µl)	2.34 ± 0.23	3.39 ± 0.35*
WBC (× 10^3^/µl)	7.17 ± 0.44	8.26 ± 0.63*
Haemoglobin (g/dL)	9.40 ± 0.61	13.56 ± 0.77*
Serum protein (g/dL)	2.66 ± 0.25	3.15 ± 0.32*
Blood glucose (mmol/L)	2.12 ± 0.26	2.15 ± 0.24

* (Asterisk) indicates a significant difference (*p < 0.05); RBC: Red Blood Cell; WBC: White Blood Cell.

#### Antioxidant activity

**Figure 1:**
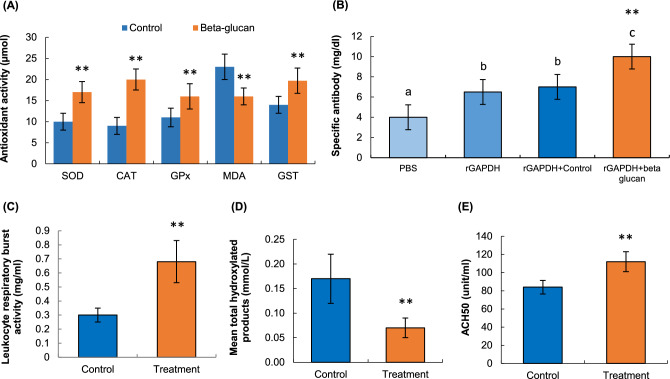
The effects of beta-glucan enriched diet on *L. rohita* as an immunity enhancer; (**A**) Dietary effects on the levels of antioxidant activity of *L. rohita*. SOD-Superoxide dismutase; CAT-Catalase; GPx-Glutathione peroxidase; MDA-Malondialdehyde; GST-Glutathione S-transferase, (**B**) ELISA for detecting specific antibody titer in *L. rohita* sera after being fed with beta-glucan. The symbols a, b, and c represent the statistical significance (**p < 0.01), (**C**) Leukocyte respiratory burst activity level of *L. rohita*, (**D**) Reactive oxygen species (ROS) activity level of *L. rohita*, (**E**) Analyses of complement activity (ACH50 assay) of *L. rohita*. All the asterisks indicate a significant difference between the groups (**p < 0.01).

SOD, GPx, and CAT activities of *L. rohita* serum increased significantly (*p* < 0.01) in fish fed with the reishi mushroom powder diet compared to the control group (Fig. 1A). GST activity was also significantly (*p* < 0.01) higher in the reishi mushroom powder powder diet group than in the control group. On the other hand, the MDA level decreased significantly (*p* < 0.01) in the treated group, indicating lipid peroxidation. However, the GST level was the highest as 19.70 ± 1.1 µmol in the treated group among the antioxidant enzymes tested and it was significantly higher than the control diet group.

#### Serum immunoglobulin

As can be seen in Fig. 1B, there was a general trend toward an increase in the production of total serum IgM in terms of specific antibody across all of the groups. There was a significant peak rise to 10 mg/dl in total serum IgM in rGAPDH + beta-glucan immunized fish as compared to controls, and this value is significantly (*p* < 0.01) higher than that in all other groups. In rGAPDH + control fish, total IgM levels were found to be statistically (*p* < 0.01) greater than in the PBS control, however, there were no noticeable changes between the rGAPDH and rGAPDH + control groups.

#### Respiratory burst activity

The leukocyte respiratory burst activity was found as 0.68 ± 0.07 mg/ml in reishi mushroom powder-treated fish, whereas in the control group, this figure made up only 0.30 ± 0.04 mg/ml, and the difference was significant (*p* < 0.01) (Fig. 1C).

#### Reactive oxygen species (ROS)

The ROS production in terms of mean total hydroxylated products in the serum of *L. rohita* was compared between the control and treated group after 4 months of feeding trial with the experimental diet of reishi mushroom powder. There was a significant (*p* < 0.01) fall in ROS production between the groups where the values were recorded as 0.17 ± 0.03 mmol/l and 0.07 ± 0.01 mmol/l in control and treatment, respectively (Fig. 1D).

#### Alternative complement activity

The effects of the supplemented diet on the alternative complement activity or ACH50 of *L. rohita* are presented in Fig. 1E. The treated group showed significantly (*p* < 0.01) greater activity in serum alternative complement when compared to the control diet group.

### Effects of bacterial infection on the immune system of dietary reishi mushroom powder treated *L. rohita*

#### Observation on behavioural and external appearances

**Figure 2:**
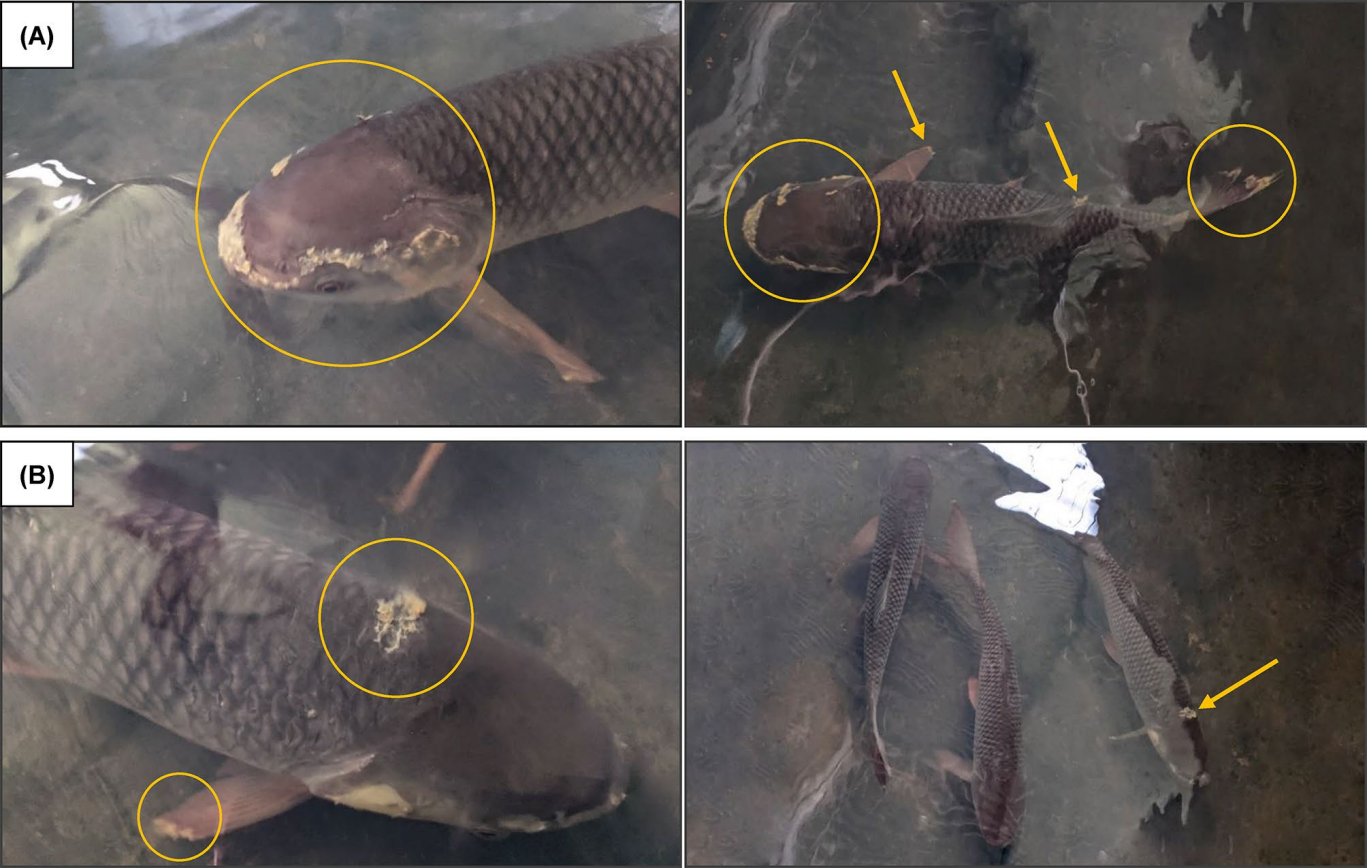
The effects of bacterial infection on the immune system of beta-glucan-treated *L. rohita*; (**A**) External infection in control *L. rohita* after the bacterial (*A. veronii*) challenge test: Infected head area and overall body, (**B**) External infection of beta-glucan treated *L. rohita* after the bacterial (*A. veronii*) challenge test: infected head and fin area as well as the overall body.

The behaviour of reishi mushroom powder-fed *L. rohita* was observed before and after the bacterial challenge test. Before exposure to bacterial suspension, there was no difference in behaviour or appearance between the control and treated fish. However, during and after the bacterial injection, several irregular behaviours such as restlessness, extreme opercula movement, imbalance swimming, and gulping were noticed in control. On the other hand, reishi mushroom powder-treated fish showed minimal aberrant behaviour. They did not show any gulping or high opercula movements, but there was a little bit of restlessness. However, a number of key changes in behaviour were noted between the experimental and control fish. The observed external infections are shown in Fig. 2. The control fish showed several skin and fin infections (Fig. 2A), whereas the reishi mushroom powder-treated fish did not show any vigorous infections on the fin, skin, and opercula (Fig. 2B). No external infection was identified in the negative control.

#### Blood serum lysozyme activity

**Figure 3:**
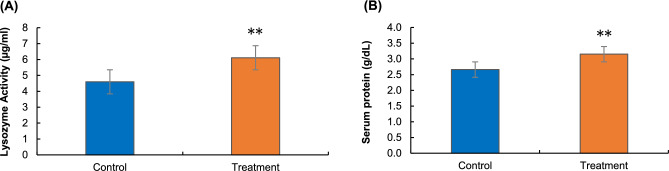
The effects of bacterial infection on the immune system of beta-glucan-treated *L. rohita*; (**A**) Lysozyme activity in blood serum in control and beta-glucan treated *L. rohita*, (**B**) Total serum protein concentration in control and beta-glucan treated *L. rohita*. All the asterisks indicate a significant difference (**p < 0.01) between treatment and control.

The blood serum lysozyme activity levels of reishi mushroom powder-treated *L. rohita* groups rose significantly (*p* < 0.01) over those of the controls after 4 months of feeding (Fig. 3A). The lysozyme activity was found to be 6.11 ± 0.89 µg/ml and 4.60 ± 0.78 µg/ml in the treatment and control groups, respectively (Fig. 3A).

#### Total serum protein

Total serum protein concentrations showed significant differences (*p* < 0.01) between the reishi mushroom powder-treated group and the control after the bacterial challenge test (Fig. 3B).

### Effects of dietary reishi mushroom powder on neurotransmitter and stress-indicating enzymes

#### Acetylcholinesterase (AChE) activity

**Figure 4:**
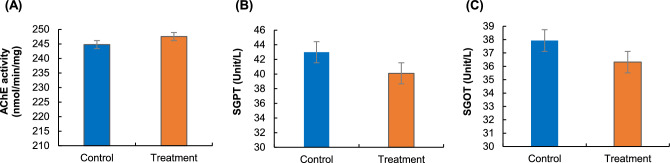
Effects of beta-glucan on neurotransmitter and stress-indicating enzyme; (**A**) AChE activity (nmol/min/mg protein) of the brain of *L. rohita*, (**B**) Serum glutamic oxaloacetic transaminase (SGOT) level in *L. rohita*, (**C**) Serum glutamic pyruvic transaminase (GPT) level in *L. rohita*. Data have been represented as mean ± S.E.

To determine AChE concentrations in the brain, *L. rohita* was given a reishi mushroom powder-enriched diet. In this experiment, AChE activity was found as 244.78 ± 2.48 nmol/min/mg in the treatment group and 247.53 ± 2.65 nmol/min/mg in the control group, which was not significantly (*p* > 0.05) different from treatment (Fig. 4A). This result indicated that reishi mushroom powder was not a source of stress to *L. rohita* during the feeding period.

#### Serum glutamic pyruvic transaminase (SGPT) and serum glutamic oxaloacetic transaminase (SGOT) level

The SGPT and SGOT of blood serum are the direct indicators of any type of stress present in the life cycle of fish. The SGPT and SGOT levels were not significantly different in control and treatment during dietary reishi mushroom powder feeding. The level of SGPT was 40.10 ± 2.2 U/ml in treatment which was also not significantly (*p* > 0.05) different from the SGPT level of 42.98 ± 1.8 U/ml in the control group (Fig. 4B). On the other hand, the SGOT level in the dietary reishi mushroom powder-treated group was 36.32 ± 2.1 U/ml, which was not statistically (*p* > 0.05) differ from the SGOT level in the control group, 37.93 ± 1.5 U/ml (Fig. 4C).

## Discussion

This study demonstrated the immuno-physiological role of dietary reishi mushroom powder on Rohu, *Labeo rohita* in improving the immune performance and tolerance against *A. veronii*. We recommended an innovative approach for enhancing immunity in aquatic farmed fish by means of functional feed additives. In the present study, an immunomodulatory dietary reishi mushroom powder-based beta-glucan (as mushroom powder contains beta-glucan) was incorporated with the regular diet of Rohu carp. Since beta-glucan cannot be synthesized in the body naturally^20^, the only way to get the compound is through outside sources such as cereal grains like barley, oats, rye, wheat, and baker's yeast, mushrooms as well. We preferred the edible reishi mushroom, *Ganoderma lucidum,* as it has a rich source of polysaccharides (beta-glucan)^2^ and other pharmaceutically active compounds (triterpenoids and proteoglycans)^4^ in its cell wall. Apart from that, it contains long or short-chain polymers of glucose subunits with beta-1,3 and beta-1,6 linkages responsible for the linear and branching structures, respectively^2^. Beta-glucan stimulates the immune system's white blood cells, increasing resistance to bacterial, fungal, and parasitic infections^21^. If a pathogenic challenge exists, beta-glucan can substitute for primary pathogen-specific receptors/sites to set off a cascade of immune system actions in white blood cells^22^. When a pathogenic microorganism attacks a fish, in terms of natural immunity non-specific processes play a greater significance than specialized responses^23^. Therefore, beta-glucan stimulates the immune system, making it more effective at defending against diseases.

The body effectively activates the specific innate immune cells, and the white blood cells get an increment in number^21,24^. In the current findings, the white blood cell count (×10^3^/mm^3^) in fish treated with dietary reishi mushroom powder showed a significantly outnumbered value compared to the control. Similarly, the number of red blood cells (×10^6^/mm^3^) was also significantly increased. These results were similar to the findings of Mahmoud^25^, Katya^26^ and Yin^6^ which indicated that the mushrooms have the ability to stimulate both the innate immune system as well as the acquired immune system^27^. Mostly in the innate defense system, beta-glucan interacts with macrophages, a category of white blood cells that is responsible for coordinating the body's defenses against infections by detecting bacteria, viruses, and other invaders^21,28^. Beta-glucan stimulates macrophages, improving their ability to recognize and eliminate pathogens. The context of this includes some methods like as phagocytosis, in which specialized cells called phagocytes to ingest and digest foreign cells or particles, and pro-inflammatory substances that eliminate infectious pathogens^29^. The macrophages then communicate with the intruders in the presence of the body's other 67 defenders^30^. After then, the acquired immune system gets involved in the fighting. When the acquired immune system receives signals from macrophages, it uses that data to produce specialized killer cells and blood components to fight off a certain challenge^23^.

Hematological parameters are considered to be the health status of fish^23^. Increases in hemoglobin concentration following the administration of beta-glucan in diet have been reported previously^2,21^, which can be a sign of stimulation of non-specific immunity^16^. Common carp, *Cyprinus carpio*, normally infested with ectoparasites, had their hematological parameters measured after being given pellets with 0.3% beta-glucan for 30 days by Şahan and Duman^30^. Hematological parameters such as hemoglobin (Hb) (g/dl), hematocrit (hct) (%), leukocyte (WBC) (×10^3^/mm^3^), erythrocyte (RBC) (×10^6^/mm^3^) number, as well as types of leukocyte cell (%), were all increased significantly (*p* < 0.05) in the fish fed with reishi mushroom powder. Mahmoud^25^ also reported that after consuming dietary white button mushrooms had a positive effect on hematological parameters (Hb, RBCs, and WBCs). They found the maximum RBCs count as 2.83 ± 0.16 (10/mm^6^), WBCs as 23.42 ± 0.33 (10/mm^3^) and Hb as 8.56 ± 0.32 (g/100 ml). Furthermore, Cruz-Garcia^31^ reported that dietary supplementation of mushroom (*Pleurotus djamor* var. *roseus*) meal at 15–20% in the diet showed a significant increase in the levels of erythrocytes, leukocytes, hemoglobin, hematocrit and lymphocyte, however, a significant decrease in neutrophil level which endorsed the findings of the present study. Nonetheless, in our study, the results could be responsible for the various compounds found from *G. lucidum*, such as beta-d-glucans, Zhi-8 proteins, and triterpenoids, and they have been studied as non-specific immunomodulating agents^5^. Therefore, using dietary reishi mushroom powder as a source of beta-glucan in the diet can result in an increased number of hematological parameters which may indicate the increased non-specific immunity in Rohu fish.

Through the formation of SOD, CAT, and GPx enzymes and the reduction of MDA level accumulation, the antioxidant response plays a significant role in scavenging excessive free radicals^21,25^. Immunostimulatory agent beta-glucan was tested on erythrocytes from grass carp that had been exposed to grass carp hemorrhage virus (GCHV) to see how it affected their superoxide dismutase (SOD) and catalase (CAT) activity^2,12^. The results revealed that beta-glucan injection increased SOD and CAT activity in fish, which is a sign of improved immunity. Similarly, the current study showed the improved antioxidant activity in Rohu fish as an indicator of improved immunity by feeding with dietary reishi mushroom powder treated feeds. This had also been proved by some findings^6,25,26^, that supplementation with dietary white button mushroom revealed increased SOD, CAT, and GPx activities in fish^25^ which is in agreement with the present study. This might be possible due to the available compounds, particularly polysaccharides and triterpenoids derived from *G. lucidum* showed evidence of antioxidant activity^4^.

This present investigation also looked at an alternate complement activity, which is part of the leucocytes' non-specific immune response^21^. Through increased complement activity, commercial beta-glucan supplements have been showed a positive effect on immunological responses^2,21^. There is a possibility that the activation of an alternative complement system is related to the protective effect of beta-glucans, as demonstrated by Yano and his group^32^. They demonstrated that pre-incubating a carp serum with glucans in vitro hindered the alternative complement system. A similar increased complement activity was observed from beta-glucan treatment by Goodridge and his team^33^. Likewise, in this study, we found that after being fed beta-glucan from the dietary reishi mushroom source, Rohu fish exhibited a significant increase in alternative complement activity. This is because *G. lucidum* contains various polysaccharides that have demonstrated the ability to increase the expression of alternative complement activity^4^ and with a subsequent favorable indication of immunological responses.

It has been established that mushroom conaining beta-glucan has an immunostimulatory impact by binding to specific receptors on the surface of defense cells^21^. These receptors cause pro or anti-inflammatory responses depending on which receptor is stimulated^30,33,34^. These defence cells include macrophages and other phagocytes, monocytes, neutrophils, dendritic cells and natural killer cells. In this study, the oral administration of dietary reishi mushroom to *L. rohita* activated receptors as indicated by enhanced immunity. Because of their importance in cellular immunity, leukocytes are often used as a biomarker for overall health. Therefore, the administration of dietary reishi mushroom (*G. lucidum*) resulted in an increase in the respiratory burst activity of the leukocytes found in *L. rohita*. There is a possibility that a greatly elevated response of monocytes and neutrophils is related to the initial response. Also, triterpenoids exhibit cytotoxic, hepatoprotective, hypocholesterolemic, and hypolipidemic properties^4^. These substances have the ability to affect the process of platelet aggregation and also act as inhibitors of angiotensin-converting enzyme and histamine release. When 1% of beta-glucan was administered to the fish, the number of monocytes increased while the number of neutrophils showed a gradual decrease. As the first line of defence against invaders, neutrophils and monocytes generate huge amounts of superoxide anion, as reported by Anderson and Siwicki^23^. A number of different fish species have shown that beta-glucan actually increases superoxide anion generation by macrophages, which is contrary to the current study^34–36^. Nevertheless, the experimental designs used in each of these studies were distinctive in the form of beta-glucan, dosage, and administration time.

Glucans with a larger molecular weight have also been shown to increase cytotoxic, phagocytic, and antibacterial activity in leukocytes, as well as the formation of reactive oxygen species (ROS)^2^. The level of reactive oxygen species (ROS) was reduced to 0.07 mmol/l in the current investigation, which is in line with the findings of Gu and Xu^37^. Beta-glucan significantly increased the hypoxia tolerance of fish compared to fish with hypoxic stress alone, showing that the beta-glucan contributed to the fish's capacity to survive the deadly hypoxic stress. In addition, reactive oxygen species (ROS) are considered a reliable biomarker of oxidative damage^38^. An increase in ROS production in stressed large yellow croakers indicated the presence of stress-induced oxidative stress^39^. When dietary reishi mushroom was provided to *L. rohita*, ROS production was greatly reduced. This suggests that beta-glucan could help repair oxidative damage to *L. rohita*. Recent studies with mushroom-based beta-glucan in virus-infected grass carp yielded comparable outcomes^12^. It's more likely that beta-glucan doses, administration times, treatment lengths, and the physiological state of the fish all have a role in the effects of beta-glucan on ROS production^2,40^. The polysaccharides found in *G. lucidum* have been observed to possess a diverse array of bioactivities, which encompass anti-inflammatory, hypoglycemic, antiulcer, antitumorigenic, and immunostimulating properties^4,5^. The prevailing consensus in scientific literature is that the antitumor and anticancer properties of polysaccharides primarily derive from their ability to bolster the immune system of the host, rather than exerting direct cytotoxic effects.

In the fish antibody, IgM, IgD, and IgT are the three major types found in fish. Among them, IgM is considered the most important one^2^. Beta-glucans appear to have long-lasting impacts on the immune system of fish when fed to the fish continuously over the course of several days. For instance, in rainbow trout, immunisation against enteric redmouth disease improved after 2 weeks of being administered beta-glucans^41^. The present study is in line with the result of this research and showed higher immunoglobulin M (IgM) in dietary reishi mushroom-treated *L. rohita*. Even after being exposed to *Vibrio alginolyticus*, grouper fed a diet for 12 days containing beta (1,4) (1,3) and beta (1,6) glucan from mushrooms exhibited increased resistance; 15 days after resuming the "control diet"^8^. Oral administration of beta 1.3/1.6 glucan (Macrogard) and immunised by anti-yersinia ruckeri vaccination, both the total number of antibody-secreting cells (ASC) and the specific levels of Ig in the serum of *Oncorhynchus mykiss* were found to be significantly higher^42^. An adjuvant effect on antibody synthesis was established by beta-glucan, when administered at a dose of 100–1000 Ig glucan/fish prior to vaccination in *Cyprinus carpio*, resulting in the production of the maximum antibody titer against *A. hydrophila*^43^. However, an increased antibody response was observed in Japanese flounder when they were fed curdlan, a beta-glucan, and formalin-killed bacteriocin *Edwardsiella tarda*. When challenged with *E. tarda* bacteriocin, the fish survival rates were much greater than in the control group^44^. Similar to our findings, Mahmoud^25^, Katya^26^ and Yin^6^ confirmed that the dietary mushroom significantly enhanced the immune status of fish species. There is a strong possibility of that because the compounds from *G. lucidum*, such as beta-d-glucans, Zhi-8 proteins, and triterpenoids, have been studied as immunomodulating agents^5^, and there is a lot of evidence that this mushroom stimulates the immune system by making cytokines and making immune effectors work better. In the present study, after the *A. veronii* bacterial challenge test, immunized Rohu showed strong prevention against the bacterial infection with a quick recovery response. These findings clearly demonstrated that an increase in IgM indicates a greater degree of immunity. Recent studies have demonstrated that *G. lucidum* has a wide range of antibacterial activity through the presence of several substances, including ganomycin and triterpenoids^4,5^. Therefore, the present research resulted in greater IgM and immunological parameters as its immunological responses.

In both marine and freshwater environments, increased innate immune responses have been achieved by using beta-glucans (1,3/1,6), through improving the leukocyte counts, and functions like complement and lysozyme activity, phagocyte activity, and respiratory burst activity^14,45^. These studies involved a wide range of fish species, for example, Nile tilapia *Oreochromis niloticus*^46^, rainbow trout *Oncorhynchus mykiss*^47^, grass carp *Ctenopharyngodon idella*^12^, sea bass *Dicentrarchus labrax*^48^, and Atlantic salmon *Salmo salar*^14^. In this study, after being fed dietary reishi mushroom-sourced beta-glucan, *L. rohita* which had been challenged by *A. veronii* showed increased serum lysozyme activity, which implies that beta-glucan has a variable effect on several immunological factors. Similarly, Yin^6^ with his team reported increased lysozyme activity after feeding the dietary mushroom *G. lucidum* on the carp’s body. Similar outcomes was observed by Wan-Mohtar et al.^9^ when the dietary reishi mushroom (*Ganoderma lucidum*) administrated on red hybrid tilapia (*Oreochromis* sp.) for sustainable aquaculture practice. On the other hand, stimulating leukocytes, as is the case with immunostimulants, boosts lysozyme activity stated by Engstad^19^. It was hypothesised from these experiments that beta-glucan improved the serum lysozyme activity in Atlantic salmon by raising the number of lysozyme-secreting phagocytes as well as the proportion of lysozyme synthesised through these cells. Dietary beta-glucan promoted phagocytosis in cells in non-stressed fish but prevented a decrease in highly stressed fish, as shown by Palić et al.^49^ in fathead minnows, *Pimephales promelas*. However, after the bacterial challenge test, the enhancement of total serum protein in *L. rohita* might well be linked to elevated complement and lysozyme levels. Surprisingly, it was observed that fish whose bodies were injected with glucan showed a significant elevation in serum lysozyme. Peptides in the blood include things like lysozyme, immunoglobulins, albumin, and complement factors, which are all components of total plasmatic protein^50^. An increase in the total protein of blood serum may be the representation of the increasing level of these serum particles. Sado^17^ described the accelerated protein concentration in blood as increased immunity in *O. niloticus* after beta-glucan administration. The present study also has found an increased serum protein value after being fed with dietary reishi mushroom. This might be possible because multiple substances derived from *G. lucidum* have demonstrated the ability to promote the proliferation and maturation of T and B lymphocytes, splenic mononuclear cells, natural killer (NK) cells, and dendritic cells^5^. Therefore, this study suggests that the dietary beta-glucan from edible reishi mushroom can increase the lysozyme activity in fish.

Throughout the cholinergic nervous system, acetylcholine (ACh) plays an important function as a neurotransmitter and neuromodulator. Some researchers believe that it has a function in vertebrates' dynamic modulation of their response to stress^51^. Possible functions of AChE in vertebrate neuro-immune systems include restoring immune system homeostasis following an infection or other inflammatory reaction^52^. Upon ingestion of pathogenic microorganisms, they are engulfed by macrophages and turned into peptides that activate the T cell to secrete cytokines. The current study did not observe any significant difference in AChE level in the brain of dietary reishi mushroom-treated *L. rohita* compared to the control. An experiment using rats fed progressively higher doses of DHA from either egg phospholipids or tuna oil yielded a similar performance. The basal release of ACh was reduced in animals fed more than 2 g DHA/kg food. Conversely, animals fed 2 or 3 g DHA/kg had a greater response to the induction of ACh^53^. Modulating cholinergic activity by diet could be possible, according to recent studies. This might occur through increased ACh release or decreased ACh hydrolysis^54^. Through the nicotinic acetylcholine receptors of effector cells, AChE can suppress the overproduction of pro-inflammatory cytokines, therefore decreasing immunological damage and reestablishing homeostasis^55^. It has been shown that long-chain PUFAs are essential for proper cholinergic transmission in *C. elegans*^56^. When pathogenic microorganisms infect the hosts, pro-inflammatory cytokines are produced in greater quantities once the innate immune response has been initiated. Due to an influx of pro-inflammatory cytokines, the afferent vagus nerve can be stimulated to release acetylcholine (ACh) at the sites of inflammation. More specifically, the ACh will control the production of cytokines by activating cholinergic receptors on immunocytes^57,58^ and attenuating inflammation. In short, vertebrate stresses can trigger the cholinergic nerve system, which in turn modulates the immune response by dampening the production of excessive pro-inflammatory cytokines and thus enhancing the innate immune system. Therefore, in this research, as unaffected cholinergic activity has been observed from the reishi mushroom treated *L. rohita*, stating that there might not have any adverse impacts associated with the dietary supplementation of beta-glucan on immunomodulation and physiology. A possible reason behind that the application of polysaccharides derived from *G. lucidum* has demonstrated the ability to increase the expression of major histocompatibility complex in a melanoma cell line. This enhancement in antigen presentation serves to further bolster the immune response against cancerous cells and viral pathogens^5^.

The parameters of SGOT and SGPT are commonly used to assess the liver and kidneys' functionality and can detect damage to these internal organs^59^. Even when all of the acclimated fish were provided supplemental food, there was no discernible change in the level of these enzymes. Nonetheless, in this study, fish fed a diet supplemented with reishi mushroom powder showed a modest elevation in these enzymes. The larvae of *Labeo rohita*^60^ and *Clarias gariepinus*^61^ also showed the same things about these enzymes fed phytogenic materials. As a result, the beta-glucan supplementation did not cause any stress to the fish during the experiment. Similarly, in the present study, supplementation with beta-glucan from the edible reishi mushroom in the diet has an impact that is conducive to enhancing the immunological responses of *L. rohita*. This could be hypothesized from that the methanolic extracts of *G. lucidum* were observed to exhibit a protective effect against cisplatin-induced kidney injury by restoring the antioxidant defense system in the renal tissue^5^. Therefore, it is evident from the current experiment that the supplementary diet associated with the reishi mushroom is stress-free and convenient. These results also suggest that the immunostimulants could be useful as a complementary or alternative method to chemo and vaccination-based preventative health care for fish populations.

## Material and methods

### Experimental design and animals

#### Fish used in this study

The Rohu carp fish (*Labeo rohita*) with both sexes used in the experiment were sourced from the Netrokona region in Bangladesh, with the help of the local fishermen. The initial length and body weight of the control fish were 43.17 ± 2.87 cm and 1042.33 ± 148.00 g, respectively. For the treatment group, the initial length was 42.81 ± 2.75 cm, and the body weight was 1022.6 ± 199.00 g. The use of experimental fish was approved by the Animal Welfare and Experimental Ethics Committee of Bangladesh Agricultural University, Mymensingh-2202, Bangladesh (AWEEC/BAU/2021). The following study was conducted in accordance with relevant guidelines and regulations. The study was carried out in compliance with the ARRIVE (Animal Research: Reporting of in vivo Experiments) guidelines.

#### Bacterial strains used in this study

The pathogenic bacteria, *Aeromonas veronii* biovar sobria was used for the bacterial challenge test in this experiment. The bacterial sample was collected from the Fish Disease laboratory under the Department of Aquaculture, Faculty of Fisheries, Bangladesh Agricultural University, Mymensingh-2202, Bangladesh. For the bacterial culture technique, selective agar media for *A. veronii* was used. Bacterial colonies were found ranging from 1.5 to 1.8 mm in diameter.

#### Ingredients used in this study

Reishi Mushroom (*Ganoderma lucidum*) was collected from Mushroom Research Center, Savar, Dhaka, Bangladesh. The supplier's analysis of the Reishi mushroom revealed that it contains beta-glucans (> 25%), total triterpenes (≥ 4.0%) and polysaccharides (≥ 20%). Additionally, an analysis was performed on the acquired Reishi mushroom powder samples. The analysis revealed that the overall crude protein content was measured at 30.41%, the total ash content was found to be 1.55%, and the total crude lipid content was determined to be 4.60%. The proximate composition of the reishi mushroom and other major ingredients in the diets are presented in Table 2.

**Table 2:** Proximate composition of the ingredients used for the feed formulation.

Indices	Fish meal	Maize meal	Rice bran	Wheat flour	Mushroom powder
Carbohydrate	10.43	54.80	59.73	62.94	41.25
Moisture	9.84	9.56	8.72	8.40	9.85
Crude protein	51.20	13.20	12.31	13.00	30.41
Crude lipid	5.05	1.05	1.00	1.05	4.60
Ash content	15.61	9.74	9.70	4.32	1.55

### Experimental method details

#### Preparation of feed and feeding trial

The mushrooms were washed and dried under the sunlight until they got a crispy texture. Then the dried mushrooms were blended in a blender machine and stored in an air-tight container for the feed preparation. Six ponds were used to study the immunological parameters, stress, and neurotransmitter effects. Six ponds having 3.7 decimal area and 5 m of depth with soil bottom were used in the research. The water quality parameters were almost similar in both of the control and treatment ponds. For the controlled feeding, we used feeding trays with exact feeding times in a day. The fish were fed twice daily, in the early morning and the afternoon at 4% body weight. Three ponds corresponded to the experimental treatment, and another three ponds were used as the control. Each pond was stocked with 20 fish from a total of 120 fish.

During the bacterial challenge test, three cisterns with two replications each were adequately cleaned, rinsed, and filled with water. A total of 24 fish (four random individuals from each control and treatment) were stocked into the two separate groups. Moreover, six additional fish from three different control ponds were used as a negative control with two replications. For bacterial administration, a suspension of *A. veronii* biovar sobria was injected intramuscularly (I/M) at a dose of 1.92 × 10^4^ CFU/ml into each of the fish from the control and treatment groups. For the negative control, the same dose of saline water (0.9% sodium chloride) was administrated to one replication of the control fish. In this study, the pathogenic bacteria, *A. veronii* dose was based on Ray et al.^11^ and Chakrabarti et al.^60^. All the fish were under feeding trial for 120 days before the laboratory and pathogenic bacterial challenge tests. For the bacterial challenge test, where the water level was maintained at 40 cm. Each cistern includes an inlet and outlet for refilling and draining. During the experiment, each cistern was also provided with a gentle shower.

Two diets were prepared with and without beta-glucan maintaining a 30% protein level (Table 3). In order to obtain beta-glucan in the treated diet, the dried mushroom powder was used (hereafter, beta-glucan diet) whereas, in the control diet, wheat flour was used instead of mushroom powder. All the dry ingredients (fish meal, maize meal, wheat flour, rice bran, vitamin B complex, and mushroom powder) were mixed according to the Pearson square method^62^ for feed formulation. When preparing the treatment diet, the ingredients were combined with reishi mushroom powder and water before being stirred, and the resulting moist dough was pelleted using a meat mincer with a diameter of 1 mm.

**Table 3:** Composition of experimental diets.

Ingredients	Treatment (%)	Control (%)
Fish meal	24.34	24.34
Rice bran	32.58	32.58
Maize meal	32.58	32.58
Mushroom	10.00	00.00
Wheat flour	00.00	10.00
Vitamin B	0.50	0.50
Total	100	100

The control feed was made into pelleted form by a similar process as treatment; the only difference was that wheat flour was used instead of mushroom powder. Then the pellet feeds were kept in trays and dried under the sunlight. After that, the formulated feeds were permitted to be kept in an airtight plastic container and maintained in a dry place at room temperature for subsequent uses.

The fish were given food twice a day, once in the morning at 8:00 A.M. and once in the afternoon (5:00 P.M.), at 4% of the body weight for 120 days. Near the designed fish shelter, fish meal was placed for consumption. The netting was done in the bottom part of the pond every 15 days at an interval in order to reduce the amount of fouling that is caused by the given food as well as metabolic products and also to supplement better-dissolved oxygen. However, 30-day periodic intervals of sampling were performed to determine the overall health of the brood fish.

#### Physico-chemical parameters of water

Every 15 days interval, records were kept of each pond's temperature, dissolved oxygen (DO), and water pH. In precision, a Celsius thermometer, electronic DO meter (multi 340 iset, DO-5509; China), handheld digital pH tester (HANNA-HI98107 pHep^®^, Romania), portable digital TDS detector (HANNA-HI98302 DiST^®^2, Romania), and a Secchi disc were used to record temperature, dissolved oxygen level, pH, Total Dissolved Solids (TDS), and transparency, respectively. Ammonia and total alkalinity were measured once in 3 days with the API^®^ ammonia and alkalinity test kit (HANNA-HI3811, Romania).

During the experimental workflow comprising the pathogenic bacterial injection operation, the temperature is one of the most crucial factors to maintain. Prior to and following the bacterial challenge test, the overall water quality of the ponds remained fairly constant. The temperature range was (28.6 ± 1.2)°C throughout the entire experimental duration and remained reasonably constant. The dissolved oxygen (DO) and pH were optimum, 6.26 ± 0.19 mg/L and 7.4 ± 0.43, respectively. The ammonia and total alkalinity were also at the optimum level for normal aquatic life. The water quality parameters are reported in Table 4.

**Table 4:** Water quality parameters during the experiment (data have been presented as mean ± standard error).

Parameters	Range (mean ± S.E.)
Temperature	28.60 ± 1.2 °C
pH	7.40 ± 0.43
Dissolved oxygen (DO)	6.26 ± 0.19 mg/L
Total dissolved solid (TDS)	99.17 ± 1.78 mg/L
Total alkalinity	120.30 ± 0.41 mg/L
Transparency	25.20 ± 1.60 cm
Ammonia	0.02 ± 0.00 mg/L

#### Hematological indices for the immune response of Rohu fish

The average length and weight for the control samples (sample size: 6) were about 44.23 ± 2.91 cm and 1164.14 ± 184 g and for the treated group (sample size: 6) were about 44.14 ± 3.05 cm and 1244.7 ± 219 g, respectively. In both the treatment and the control groups, blood was taken through the vein of the caudal peduncle from fish and stored in eppendorf tubes in an icebox. Tri-sodium citrate (3.8% w/v) was used as an anticoagulant during the collecting of blood from both of the samples. Immediately after that, the collected blood was placed inside the centrifuge machine at 4 °C temperature. Following centrifugation at 8000 rpm for 15 min, the serum was stored at 4 °C in a 1.5 ml Eppendorf tube for subsequent hematological analysis.

Blood parameters such as WBC and RBC count, hemoglobin concentration, glucose concentration, and total serum protein concentration were measured as part of the hematological analyses. The level of glucose in the blood was measured with the help of a glucometer (Health Assure^®^, Taiwan), and hemoglobin (Hb) was determined using hemoglobin strips (Easy Mate^®^ GHb; Bioptik Technology Inc., Miaoli County 35057, TAIWAN PRC).

For RBC and WBC count, 5 μl blood was diluted in 995 μl RBC solution, and another five μl blood was diluted in 195 μl WBC solution, respectively, and were placed on a hemocytometer immediately after collection. Blood samples were examined to estimate the numbers of erythrocytes (RBC × 10^6^/mm^3^) and leucocytes (WBC × 10^3^/mm^3^) using a research microscope (OPTIA B-350, Italy) at 40× magnification. The following formulas were then used to estimate the blood cells:

$$\mathrm{RBC }(\times {10}^{6}/{\mathrm{mm}}^{3})=\frac{\mathrm{Total \; cells \;no}. \; \mathrm{ in } \; 5 \;\mathrm{ large \;squares}\times \mathrm{dilution\; factor}\times \mathrm{depth\; factor}}{\mathrm{No}. \; \mathrm{ of \;small\;  squares \;counted }\times 16}$$

$$\mathrm{WBC }(\times {10}^{3}/{\mathrm{mm}}^{3})=\frac{\mathrm{Total \;no}. \; \mathrm{ of \; cells \; in }\;1\;\mathrm{ large \;squares}\times \mathrm{dilution \;factor}}{\mathrm{Volume \;factor}  \; (0.1)}$$

### Determination of antioxidant activity

#### Superoxide dismutase (SOD), catalase (CAT) and glutathione peroxidase (GPx) activities

The enzyme activities of the liver were evaluated by utilizing commercial kits (Cell Biolabs Inc., San Diego, USA) to analyze superoxide dismutase (SOD), catalase (CAT), and glutathione peroxidase (GPx) activity. For each gram of liver, 5–10 ml of cold 1X lysis buffer (10 mM Tris, pH 7.5, 150 mM NaCl, and 0.1 mM EDTA) was used to create a homogenous mixture. To get the tissue lysate supernatant, we centrifuged the homogenized material at 12,000 rpm for 10 min. Following this, the 96-well plate was prepared by pipetting the SOD sample, chromogen solution, 10X SOD assay buffer, and de-ionized water into the appropriate wells as directed by the manufacturer. Thereafter, each well received 10 µl of pre-diluted 1X xanthine oxidase solution and was incubated for 1 h at 37 °C. A microplate reader was used to measure the absorbance at 490 nm. Pipetting was used to transfer 25 µl of catalase standards, controls, and samples into each individual well of the microtiter plate that was used for the catalase test. The microplate with the standards, samples, and controls had another 25 µl of the 40 µM hydrogen working solution poured into each well. After that, the reaction mixture was let to sit in an incubator for half an hour at room temperature. After adding standards, controls, and samples to individual wells, 50 µl of the (10-Acetyl-3, 7-dihydroxyphenoxazine) ADHP/Horseradish peroxidase (HRP) working solution was added to each one before being incubated on a shaker for half an hour at 37 °C in a light-proofed way. For this experiment, we used a fluorescence microplate reader with excitation in the 530–570 nm range and emission in the 590–600 nm wavelength range to determine the absorbance. A total of 25 µl of the 1X nicotinamide adenine dinucleotide phosphate (NADPH) solution was poured into each well of the 96-well plate to facilitate the glutathione peroxidase reaction. Following that, each well received 100 µl of the produced glutathione peroxidase standards or samples, followed by 50 µl of the 1X chromogen. After adding 25 µl of the glutathione disulfide solution, the absorbance was checked by each minute for 10 times at 405 nm wavelength. There, the concentration of the enzyme needed to oxidize 1.0 µmol of NADPH to NADP+ per minute at 25 °C is referred to as one unit.

#### Glutathione S-transferase (GST)

According to the guidelines provided by the manufacturer, the GST activity was assessed using a GST Commercial Kit (Colorimetric, ab65326, Abcam plc, Cambridge, UK). Heparin (0.15 mg/ml) was added to phosphate-buffered saline (PBS) to eliminate blood cells and clots before it was used to wash liver tissue isolated particularly for GST. Next, 500 µl of GST assay buffer was used to re-suspend the sample. The combined ingredients were then homogenized in a mixer before being centrifuged at 10,000 rpm for 15 min under 4 °C temperature. The resulting supernatant was then collected in a clean tube. Each well had 10 µl of sample and positive control, 40 µl of GST assay buffer, and the "negative control" well had 50 µl of GST assay buffer. Both the sample and the control wells received an addition of 5 µl of glutathione. In order to get the reaction started, the plate was gently agitated while each sample well and the control sample well were each given 50 µl of the reaction mixture. At a wavelength of 340 nm, the kinetic mode of a microplate reader was utilized to take the absorbance reading, which was taken at intervals of 2–3 min for at least 10 min in a light-proofed room temperature environment.

#### Malondialdehyde (MDA)

The procedure that was developed by Jain^63^ was utilized in order to determine the MDA content of liver tissues. In short, thin-layer chromatography (TLC) can measure how much MDA is cross-linked between phosphatidylethanolamine and phosphatidylserine. This method has also been used to measure the damage caused by lipid peroxidation. The lipids were extracted, dried, and washed in accordance with the procedures described by Rose and Oklander^64^. TLC used a solvent system consisting of 50% chloroform, 25% methanol, 8% glacial acetic acid, and 4% water to separate different types of phospholipids in the lipid extract on silica gel H glass plates (silica 60, 0.25 mm thick, Brinkman, Westbury, NY). When the TLC plate was exposed to iodine vapours, the various phospholipid spots were visible, and a fine needle was used to encircle them. Authentic standards produced constantly from haemoglobin A at a pace based on the ambient glucose content substantiated the localization of different phospholipids on the TLC plate. However, there are no enzymes involved in the synthesis of GHb, which means that it is a gradual and permanent process. The measurement of GHb in a single blood sample is now generally recognized as a reference for the previous day's mean blood glucose level. After blood samples were subjected to biochemical analysis in a clinical laboratory, GHb values were determined.

#### Determination of respiratory burst activity

The respiratory burst activity of the leukocytes was measured by immediately processing the blood after collection. The experiment was conducted with minor modifications to the methodology developed by Anderson and Siwicki^23^. The reactive oxygen species (ROSs) generated by the respiratory burst of leukocytes are measured by a colourimetric assay that involves the reduction of nitroblue tetrazolium (NBT, Sigma, St. Louis, MO, USA) into a dark blue precipitate (formazan granules) within the phagocyte. Following the bleeding of the fish, 100 µl of 0.2% nitroblue tetrazolium solution (NBT, Sigma, St. Louis, MO, USA) were combined with 100 µl of heparinized blood. After that, a half-hour incubation phase was carried out at a temperature of 24 °C immediately following the homogenization of the final solution. The phosphate-buffered saline (PBS) used in the preparation of the NBT solution which had a pH of 7.4, and it contained the components in the following concentrations: KCl (2.7 mM), NaCl (0.137 M), Na_2_HPO_4_ (8.1 mM), CaCl_2_ (0.9 mM), KH_2_PO_4_ (1.5 mM), and MgCl_2_ (0.49 mM) in distilled water Milli-Q qsp 1 L. In a glass tube containing 1 ml of N, N-dimethyl formamide (DMF, Sigma, St. Louis, MO, USA), 50 µl of the solution was mixed after incubation and the second round of homogenization. Following the homogenization, the resulting solution was then centrifuged for 5 min at 3000 rpm. By using a spectrophotometer (Beckman DU-70S), we measured the optical density (OD) of the supernatant at 540 nm wavelength. In addition, the blood was replaced with distilled water in the blank, but apart from that, everything else was the same.

#### Determination of reactive oxygen species (ROS) of blood

The three products (catechol, 2,3 dihydroxy benzoic acid, and 2,5 dihydroxybenzoic acid) of hydroxyl radical attack on salicylic acid were quantified and separated using high-performance liquid chromatography (HPLC). This method was originally evolved from Owen^15^ and was later authenticated by Orozco^65^. Firstly, standards of salicylic acid with the above-mentioned three products were used to develop a calibration plot. After that, a 100 µl blood sample was undertaken for 21 h of incubation period at 37 °C temperature. During this incubation, 50 µM FeCl_3_H_2_O, 500 mM ethylenediaminetetraacetic acid (EDTA), 2 mM salicylic acid, and 100 mM phosphate buffer solution were used. Each sample was filtered to remove any contaminates after incubation, and then 20 µl was loaded into the HPLC column (ODS Hypersil 200 × 2.1 Thermo Fisher Scientific Inc.). The mobile phase consisted of 2% glacial acetic acid in water (solvent A) and methanol (solvent B) for chromatographic compound separation. The chromatographic separation employed the following gradient: 95% A/5% B for 2 min, 75% A/25% B for 8 min, 60% A/40% B for 10 min, 50% A/50% B for 10 min, and 0% A/100% B for 10 min. The UV/VIS detector was initially set at 278 nm and then switched to 301 nm for the remaining 5.5 min of the operation time. Using the ChemStation program (Agilent ChemStation, Agilent Technologies, Santa Clara, CA, USA), we were able to manage our data and operate our instruments at the appropriate flow rate of 0.5 ml/min.

#### Determination of specific IgM detection by ELISA

The specific IgM in Rohu's blood serum was estimated with minor modifications using Tang's^18^ procedure. In brief, each microplate reader well was coated with 20 µg rGAPDH in 100 µl of carbonate buffer three times. Then the well was washed with PBST (phosphate-buffered saline with Tween^®^ detergent) and blocked with 4% BSA-containing PBS buffer. After obtaining the blood from the caudal peduncle, a centrifuge was used to separate the serum from the blood. The serum was diluted twofold with PBS and carried out in 60 min incubation period at 37 °C temperature. Then, 100 µl of goat anti-mouse Ig-alkaline phosphate and 100 µl of anti-fish IgM monoclonal antibody were given per each well, and the mixture was further incubated at 37 °C for 60 min. In order to develop the colour, pNPP substrate was added, and the plate was read at 405 nm in an ELISA reader. The highest dilution of serum giving double OD compared to the control serum was considered as the ending point of the antibody titer of serum. The titer geometric mean was expressed as a reciprocal log_2_ value of the highest dilution of all treatment samples.

#### Analysis of complement activity (ACH50 assay)

Following the approach of Rakhi^66^ with slight adjustments, complement activity (ACH50) was measured in whole blood homogenates (WBH) and serum by utilizing sheep (SRBC) and rabbit (RRBC) red blood cells. Heat-inactivation at 56 °C for 30 min was used on five samples of guppy WBH and rabbitfish serum as a negative control. For each analysis, 30 μl of red blood cells (2.5 × 10^8^ cells/ml for WBHs, and 4 × 10^8^ cells/ml for serum) were combined with 100 μl of serially diluted serum/WBH in working buffer (dilutions ranged from 1:16 to 1:1024 for gelatin veronal buffer and EGTA Mg^2+^ GVB). Double distilled water (DDW) was utilised for complete lysis, in the same way, that the working buffer was combined with the red blood cells in the blank condition. After adding in the catalyst, we kept the reaction mixtures incubated for 2 h at room temperature (24 °C) with gentle stirring. The hemolytic process, on the other hand, was halted by the addition of 1 ml of stop solution (10 mM EDTA working buffer). To separate the components, the solutions were then centrifuged at 4 °C for 10 min at 1600 rpm. Red blood cell (RBC) lysis in the reaction was then optically assessed by checking whether or not there were any RBC particles in the tube and observing for any noticeable change in its size. We next used a Tecan Sunrise™ spectrophotometric method (Salzburg, Austria) to measure the OD at 414 nm after transferring 200 μl of the reaction supernatant to a 96-well microplate. The ACH50 titer was defined as the reciprocal of the diluted sample that caused 50% RBC lysis where values are represented in units per millilitre. In this case, we solely employed a visual inspection of the pellet before taking an OD reading to double-check our results.

#### Exposure to bacteria for a challenge test

At the end of a 4-month feeding trial, experimental *L. rohita* was challenged against the pathogenic bacteria *Aeromonas veronii*. For this, four individuals from each of six ponds were randomly selected and kept in separate cisterns (each having two replications) for the bacterial challenge test. For bacterial administration, a suspension of *A. veronii* was injected intramuscularly at a dose of 1.92 × 10^4^ CFU/ml into each of the fish from the control and reishi mushroom treated groups. For the negative control, the same dose of saline water (0.9% sodium chloride) was administrated to one replication of the control fish. Fish were released into cisterns that were either treated with an experimental infection or served as a control. The fish that were affected in the experiment were monitored for 7 days. Within the experiment, the fish were kept in a tank with constant aeration and were not fed at any point. In addition, every day, a third of the tank's water was changed and any waste was removed using a syphon. Finally, the presence of infection was documented through the examination of the mouth and fin, ulcers, clinical appearances, as well as death rates.

#### Determination of lysozyme activity

The turbidimetric method of Litwack^67^ was used to measure the amount of lysozyme in blood plasma. *Micrococcus lysodeikticus* bacterium standard solution was prepared by adding 0.6 mg of *M. lysodeikticus* for every millilitre of phosphate buffer (0.05 M Na_2_HPO_4_⋅2H_2_O: disodium hydrogen phosphate dihydrate, in 1000 ml distilled water; pH 6.2). A 100 µl of blood serum was taken into a cuvette on a spectrophotometer set at 450 nm. The cuvette was filled with 650 µl of *M. lysodeikticus* solution. After 30 s of combining the bacterial solution and serum, the initial absorbance reading was taken. The absorbance was recorded for 3 to 5 min at 30-s intervals. The absorbance was put on the standard curve's Y-axis, and the lysozyme concentration was calculated by back-calculation. The standard curve was plotted by Chicken egg lysozyme activity against the bacterial solution. The powdered form of chicken egg lysozyme (Sigma Chemical Company, St. Louis, USA) was diluted into a stock solution of 40 ml by adding 1 mg of lysozyme powder to the buffer. The following standard solutions were used: 1, 2, 4, 5, 8, and 10 µg/ml). The standards were reacted with the bacterial solution. A standard curve was plotted to put the optical density along the Y-axis, with the standard concentration along the X-axis.

#### Determining serum total protein concentration

After collecting blood, it was centrifuged at a temperature of 4 °C. for 10 min at a speed of 10,000 rpm. The total serum protein concentration estimation was proceeded following the procedure of Lowry et al.^68^, in which a reference standard of bovine serum albumin in a homogenization buffer was used. Briefly, 0.01 ml of 2 N NaOH was mixed with 0.01 ml tissue homogenates and complex forming reagent (2% Na_2_CO_3_, 2% C_4_H_4_O_6_KNa·4H_2_O and 1% CuSO_4_·5H_2_O in the proportion of 100:1:1). After that, the solution was provided 0.1 ml of Folin reagent then mixed with the vortex mixture, and permitted to stay for 30 min at room temperature. A spectrophotometer (SPECTRONIC GENESYS™5) was used to measure absorbance shifts at 750 nm wavelength. Bovine serum albumin was utilised as the standard to create an absorbance standard curve which was then used to estimate the concentration of the sample.

#### Determination of the neurotransmitter, AChE activity

*Labeo rohita* was dissected for AChE activity analysis, and the entire brain was taken and kept in an ice-cold 0.1 M sodium phosphate buffer solution (8.0 pH). We next used a glass-teflon homogeniser to break down the tissues in a homogenization buffer (0.1% Triton X-100, 0.1 M sodium phosphate buffer with a pH level of 8.0) in order to get a phosphate buffer concentration of 20 mg tissue/ml. Protein concentration was determined by centrifuging the tissue homogenate at 4 °C temperature for 10 min at 2000 rpm; discarding the supernatant, and then following the protocol established by Lowry et al.^68^. In which, as a standard, bovine serum albumin in homogenisation buffer is utilised. Minor modifications were made to Ellman et al.^69^ approach for measuring AChE activity in the fish brain. Firstly, 50 µl of tissue homogenate and 50 µl of 5,5-dithobis (DTNB: 6 mM, 2-nitrobenzoic acid) were mixed with 900 µl of ice-cold 0.1 M sodium phosphate buffer (pH value: 8.0, 0.1% Triton X-100). After that, the mixture was given a proper vortex and let to stay about 10 min at room temperature. Thereafter, we transferred triplicate 200 µl aliquots in microtitre plate wells. 50 µ of fish-specific acetylthiocholine iodide (15 mM AtChI) were added to initiate the reaction. The microplate reader (Model: SPECTRA max 340PC384) was used to monitor the absorbance at an interval of 12 s for 10 min at 412 nm wavelengths. The percentage was computed by the following equation: (We measured AChE activity in terms of nmol/min/mg of protein)

$$ {\text{R}} = { 5}.{74 }\left( {{1}0 - {4}} \right) \, \Delta {\text{A}}/{\text{C}}_{0} $$

where R = substrate hydrolysis rate in moles per minute per gramme of tissue; C = the initial tissue concentration; ∆A = absorbency per minute-scale shift.

#### Determination of plasma enzymes activity

An adaptation of the method developed by Reitman and Frankel^70^, which was considered to determine SGOT and SGPT activities in this study. After taking a fresh blood sample, it was then centrifuged for ten minutes at a speed of 11,000 rpm. Two 15-ml screw-cap test tubes were filled with one ml each of SGOT buffer (50 ml of phosphate buffer, 0.89 g of alanine, and 0.0146 g of α-ketoglutarate) and SGPT buffer (50 ml of phosphate buffer, 1.33 g of aspartate, and 0.0146 g of α-ketoglutarate). The buffer solution then underwent a 10-min incubation period at a temperature of 40 °C. Both buffers had two ml of serum added to each and then vortexed to combine. An hour of time was used to incubate the combination at 40 °C temperature for SGOT, while half an hour was used for SGPT. Following incubating for 20 min at room temperature after adding 1 ml of 2,4 di-nitrophenyl hydrazine, the mixture was filtered. Each solution received 10 ml of 0.4 N NaOH, which was then inverted and stirred for 30 min. Finally, at a temperature of 24 °C, the optical density was measured at a wavelength of 505 nm, where the measurement units for both variables were U/ml.

#### Data analysis

Using the SPSS v26 computer programme, the statistically significant difference in the mean between the control and treatment groups was assessed by using a t-test analysis program. mean ± standard error (S.E.) were used to summarize the data, and statistical significance was determined at a p-value of 0.01 or 0.05.

## Data Availability

The lead contact, upon request, will share all data reported in this paper. This paper does not report the original code. This study did not generate new unique reagents or genetic sequences. Any additional information required to re-analyse the data reported in this paper is available from the lead contact upon request.
